# Radiomics for predicting sensitivity to neoadjuvant chemotherapy in osteosarcoma: current status and advances

**DOI:** 10.3389/or.2025.1633211

**Published:** 2025-10-17

**Authors:** Panhong Zhang, Weitao Yao, Zhehuang Li, Yichao Fan, Xinhui Du, Bangmin Wang, Fan Zhang, Jingyu Hou, Qilong Su

**Affiliations:** ^1^ Henan Cancer Hospital Affiliated Cancer Hospital of Zhengzhou University, Zhengzhou, China; ^2^ Zhengzhou University, Zhengzhou, China; ^3^ Zhoukou Central Hospital, Zhoukou, China

**Keywords:** osteosarcoma, chemotherapy, response, prediction, radiomics, deep learning

## Abstract

Osteosarcoma is the most common primary malignant bone tumor, accounting for approximately 20% of all primary malignant bone tumors, and predominantly affects adolescents. The current standard treatment involves a multimodal approach combining neoadjuvant chemotherapy, surgical resection, and postoperative adjuvant chemotherapy. However, patient responses to chemotherapy vary significantly, with response rates (defined as patients achieving ≥90% tumor necrosis) ranging from 30% to 60%. Chemotherapy sensitivity is one of the most critical prognostic factors, and this heterogeneity underscores the importance of predictive tools for optimizing individualized treatment and improving clinical outcomes. In recent years, radiomics has emerged as a revolutionary paradigm in medical imaging analysis. By extracting high-throughput, deep-layer feature information from medical images, it provides a novel technical pathway for quantitative tumor phenotyping. Advanced computer vision algorithms enable the automated extraction of thousands of quantitative metrics—including morphological (shape features), intensity (first-order statistics), and texture (second- and higher-order features)—from multimodal imaging data such as Computed Tomography (CT), Magnetic Resonance Imaging (MRI) and 18F-Fluorodeoxyglucose Positron Emission Tomography (18F-FDG PET/CT) These features not only precisely characterize tumor heterogeneity and the microenvironment but also overcome the subjectivity and reproducibility limitations of traditional manual image interpretation. Leveraging these advantages, radiomics has demonstrated significant value in predicting neoadjuvant chemotherapy efficacy in osteosarcoma.

## Introduction

Osteosarcoma (OS), the most common primary malignant bone tumor, accounting for approximately 20% of all primary malignant bone tumors, primarily occurs in children and adolescents, with the metaphysis of long bones being the typical site of involvement (1). Modern treatment strategies, including optimized neoadjuvant chemotherapy (NAC) protocols and comprehensive therapeutic approaches, have improved the 5-year survival rate to 60%–70%. The current clinical standard employs multi-agent chemotherapy regimens, such as high-dose methotrexate (HD-MTX), ifosfamide (IFO), doxorubicin (ADM), and cisplatin (DDP) (2). However, significant heterogeneity in patient responses to the same regimen highlights the need for predictive systems to assess chemotherapy sensitivity. The EURAMOS-1 trial found only approximately 50% of patients achieve good histological response (defined as ≥90% tumor necrosis after neoadjuvant chemotherapy). This response rate has important prognostic implications, as it guided treatment stratification in the trial, underscoring the need for predictive tools to optimize chemotherapy selection and improve outcomes (3, 4).

The clinical value of chemotherapy response prediction spans three key dimensions. Firstly, at the level of treatment optimization, accurate predictive models enable truly personalized therapeutic strategies. For patients likely to respond well to chemotherapy, treatment can be optimized (e.g., maintaining effective regimens to avoid unnecessary escalation), while those predicted to be poor responders can be promptly transitioned to alternative therapies (currently under investigation in clinical trials, such as targeted agents or immunotherapy). Secondly, in terms of prognostic management, reliable prediction tools facilitate early identification of high-risk patient populations, allowing for more intensive monitoring and timely clinical interventions. Finally, from a research translation perspective, robust prediction systems provide valuable stratification criteria for clinical trial design. For example, in sarcoma trials like SARC024 (5), predictive tools have enabled stratification of patients into novel therapy arms based on predicted resistance, accelerating the drug development process and improving clinical research efficiency (2, 6).

Therefore, the development of reliable tools to predict chemotherapy sensitivity is critically important in clinical oncology. Currently, the standard method for assessing NAC response in osteosarcoma relies on histopathological evaluation of tumor necrosis in surgical specimens, as described by Huvos et al (7). Patients with over 90% tumor necrosis are classified as good pathological responders, while those with less necrosis are considered poor pathological responders. However, this approach can only be applied after surgery, making it unsuitable for early prediction before or during treatment. To date, no definitive clinical, biological, or imaging markers allow clinicians to reliably predict chemotherapy response early enough to modify treatment strategies (8).

Notably, “response to chemotherapy” encompasses two distinct concepts in osteosarcoma: histological response, defined by post-surgical tumor necrosis, and radiological response, reflected by imaging changes (e.g., tumor size or density alterations on CT/MRI). Radiomics-based predictive tools primarily aim to predict histological response using pre-treatment imaging, bridging the gap between early radiological assessment and delayed surgical pathology (8, 9). Existing radiological criteria such as RECIST are less applicable to osteosarcoma due to the presence of bone matrix, which complicates assessment of tumor size changes. This limitation highlights the need for radiomics, which can extract subtle features beyond conventional size-based metrics (10, 11).

Radiomics offers a promising alternative for predicting NAC response, particularly in osteosarcoma. Unlike conventional methods, radiomics can integrate data from multiple imaging modalities—including Computed Tomography (CT), Magnetic Resonance Imaging (MRI) and ^18^F-Fluorodeoxyglucose Positron Emission Tomography (^18^F-FDG PET/CT) to provide a comprehensive assessment of tumor structure and function. Its noninvasive nature also allows for repeated monitoring throughout therapy. Furthermore, radiomics can extract subtle imaging features beyond visual detection, potentially uncovering biomarkers of treatment response (12–14). When combined with clinical and genomic data, these imaging features may improve predictive accuracy (15).

The development of predictive models from imaging data relies on artificial intelligence (AI)—a broad umbrella concept encompassing algorithms designed to mimic human cognitive functions (16). Within AI, machine learning (ML) represents a core subset, focusing on algorithms that learn patterns from data to make predictions without explicit programming (17). A specialized branch of ML, deep learning (DL), utilizes multi-layer neural networks to automatically extract hierarchical features from complex data (e.g., medical images), enabling it to process high-dimensional information more effectively than traditional ML methods (17, 18). Conventional statistical methods demonstrate significant limitations when processing the high-dimensional, nonlinear data characteristic of radiomics, particularly in their restricted capacity for feature extraction and limited ability to recognize complex patterns (13). In contrast, convolutional neural networks (CNNs)—a type of DL algorithm—utilize multi-layer convolutional kernels with localized receptive fields and parameter-sharing properties to automatically extract hierarchical image features. This approach enables simultaneous analysis of both microscopic texture characteristics and macroscopic morphological patterns (6, 14). The end-to-end learning framework provides two distinct advantages: first, it eliminates the selection bias inherent in manual feature engineering; second, through backpropagation optimization, it can directly identify potential imaging biomarkers predictive of chemotherapy response from raw pixel data (6, 14). Clinical validation studies have established that deep learning-based prediction models demonstrate superior performance in assessing neoadjuvant chemotherapy response (19, 20).

Emerging evidence demonstrates that combining conventional imaging features [CT/MRI (19)] with functional parameters [DWI/DCE-MRI/^18^F-FDG PET (9, 21)] using artificial intelligence algorithms can establish highly predictive NAC response assessment systems. This multimodal integration approach has shown clinical value in predicting treatment response across various malignancies, offering new possibilities for precision therapy in osteosarcoma (14, 22).

This comprehensive review followed Preferred Reporting Items for Systematic Reviews and Meta-Analyses (PRISMA) guidelines to systematically evaluate studies investigating radiomics for predicting NAC response in osteosarcoma. We performed quantitative meta-analysis using random-effects models to compare the predictive performance of different imaging modalities (MRI,PET-CT), calculating pooled sensitivity, specificity, and area under the receiver operating characteristic curve (AUC). Study heterogeneity was assessed using I^2^statistics. Furthermore, we critically analyzed key factors affecting prediction accuracy, including feature selection methods, machine learning algorithms, and validation strategies, to provide evidence-based recommendations for clinical practice. The results highlight MRI’s superior capability in multimodal data integration and high-throughput feature extraction. These findings provide robust evidence to support individualized treatment decision-making in clinical practice.

## Methods

### Literature retrieval and study selection

To comprehensively identify studies on radiomics-based prediction of NAC response in osteosarcoma, we conducted a systematic literature search following PRISMA guidelines. Our search strategy encompassed three major databases for articles published between 1 January 2014 and 31 December 2024. We employed the following search terms: “osteosarcoma AND chemotherapy AND response AND prediction” to ensure precise identification of relevant studies. The search was restricted to English-language original research articles, excluding review articles.

Two independent investigators (Zhehuang Li and Panhong Zhang) performed initial screening based on titles and abstracts. Potentially eligible studies underwent full-text review. Any discrepancies were resolved through consensus discussion.

Studies were included if they met the following criteria: (1) histologically confirmed primary osteosarcoma; (2) evaluation of neoadjuvant chemotherapy (NAC) response prediction; and (3) reported pathological response (e.g., ≥90% necrosis). Exclusion criteria comprised: Studies not reporting relevant outcomes; Research not including the specific term osteosarcoma; Investigations limited to post-NAC assessment only; Prognostic rather than predictive studies; Non-imaging based approaches.

**FIGURE 1:**
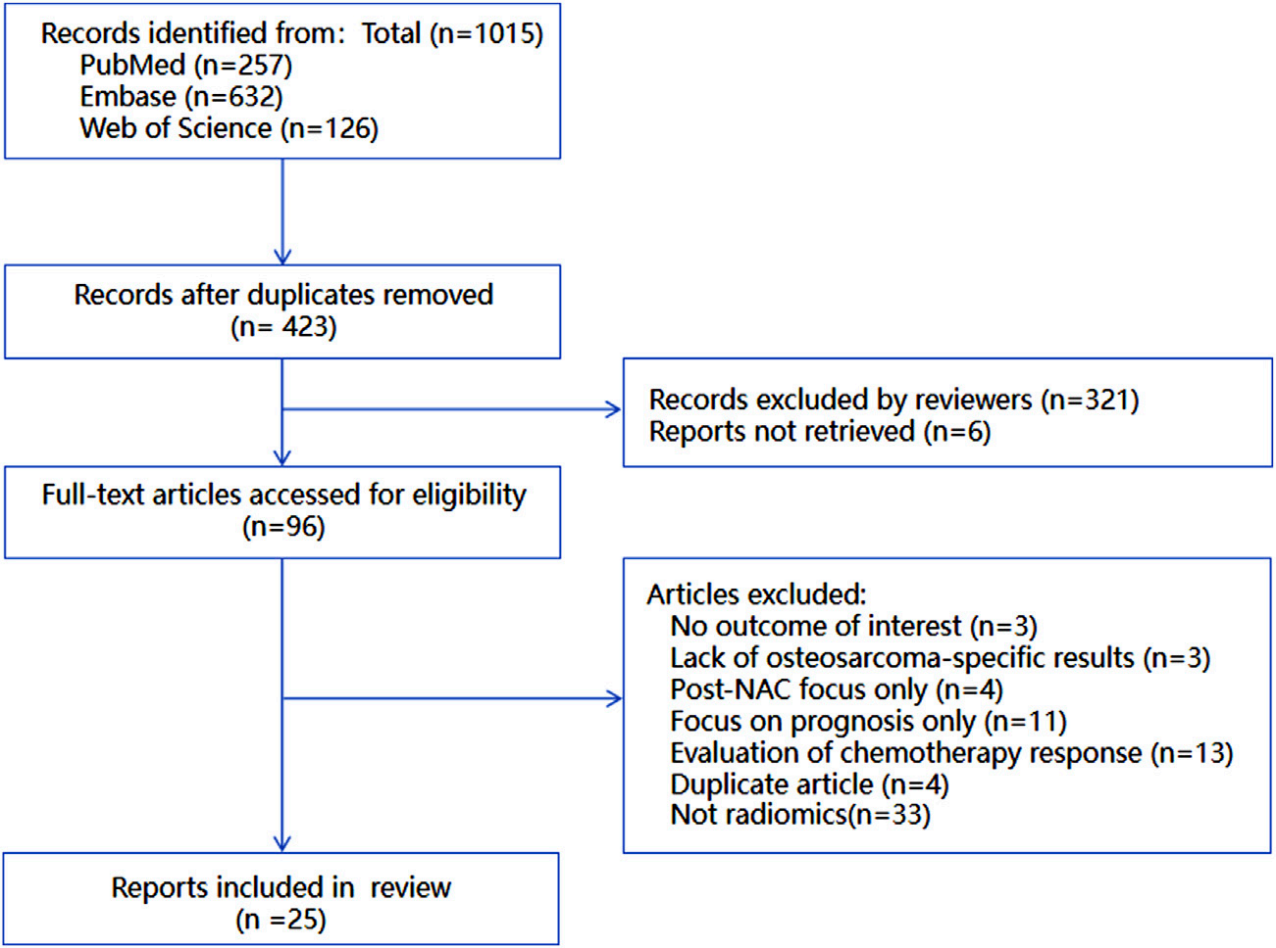
Flowchart of the study-selection procedure.

The complete study selection process is illustrated in Figure 1, demonstrating rigorous application of our predefined criteria to ensure methodological quality and relevance to the research question.

### Data extraction

Two investigators independently extracted data from the included studies using a standardized form designed to capture essential study characteristics. The extraction template documented publication year, study design, author information, patient demographics, imaging parameters, radiomic feature extraction methodology, predictive model construction, and primary outcomes (Table 1). When encountering missing or ambiguous data, we attempted to contact the original authors for clarification. Any unresolved data limitations were explicitly acknowledged in our analysis.

**TABLE 1:** Characteristics of the 24 studies included in the present review.

Imaging method	Author	Year of publication	Patients	Centers	Image segmentation	Radiomics information extraction	Prediction model building	General result
X-ray films								
X-ray and MRI	Z. Luo (23)	2023	N = 102	1	Manual segmentation (ITK-SNAP marks ROI)	Pyradiomics package extract features from ROI Intra-group correlation coefficient (ICC) (ICCs >0.75):filter features mRMR and LASSO regression:feature selection	LR	Models using clinical, X-ray radiomics, MRI radiomics, X-ray plus MRI radiomics, and all data combined had AUC values of 0.760, 0.706, 0.751, 0.796, and 0.828, respectively
CT images								
CT	F. Yang G (24).	2024	N = 225	2	Manual segmentation (ITK-SNAP marks 3D VOI)	Radiomics features: intensity statistics, geometric features, texture features, etc	LASSO、LR、Nomogram	Models combining radiomics and clinical features had AUC values of 0.78 and 0.75 in the training and independent validation sets, respectively
CT	D. Fu (25)	2023	N = 18	1	Manual segmentation	MIMICS 20.0 to extract the following parameters: 1. Peri-osteosarcoma fat attenuation index (FAI) 2. Periosteosarcoma fat volume (PFV) 3. Fat to volume ratio (FVR)	LRA	The area under ROC curve (AUC) of FAI and 6-h MTX concentration were 0.950 and 0.963, respectively, indicating that they had good predictive performance in predicting chemotherapy response
CT	L. Xu (15)	2021	N = 157	1	Manual segmentation (ITK-SNAP outline the tumor)	Based on the open source Radiomics package in MATLAB 2017b:the tumor area; the tumor area	LOOCV、mRMR、Multivariate logistic regression algorithm	This paper improves the accuracy of prediction of neoadjuvant chemotherapy response in osteosarcoma patients by combining CT radiomic features of tumor and non-tumor bone areas and using multiple machine learning techniques
(99m)Tc-MIBI	C. Wu (26)	2019	N = 30	1	Manual segmentation (ITK-SNAP marks ROI)	Tumor to background ratio (T/B), tumor MIBI washout rate (WR), change rate of tumor uptake after chemotherapy (AR)	0	In pre-chemotherapy MIBI imaging, tumor washout rate (WR) was negatively correlated with tumor necrosis rate (r = −0.510, P = 0.004). When WR ≤ 25% was used as the threshold for predicting good chemotherapy response, the sensitivity was 100%, the specificity was 91.7%, and the accuracy was 95.8%
MRI								
axial T2WI and T1CE	F. Zheng (27)	2024	N = 106	1	Auto3DSeg framework: DiNTS、SegResNet two segmentation models	The first-order statistics, shape features, and texture features are used to extract features from the transformed image through a variety of filters	DLR:ResNet18、LR、MLP; LASSO; SVM	the DLR model achieved the highest prediction performance with an accuracy of 93.8% and an AUC of 0.961 in the test sets
T2WI and T1CE (a single MRI sequence at a single time point)	Y. Zhang (20)	2024	N = 109	1	Manual segmentation (ITK-SNAP marks VOI)	Radiomics features of T2WI and T1CE	RF、 LR、DCA	The combined model (the pre-NAC, post-NAC)achieved the highest areas under the receiver operating curve (AUC) values of 0.999 and 0.915 in the training and test sets, respectively. The AUCs of the post-NAC model were higher than those of the pre-NAC model
baseline MRI	Kanthawang, Thanat (11)	2024	N = 95	1	Manual segmentation (VOI)	Multiple parameter combinations:tumor volume, maximum axial diameter, necrotic area, and extent of soft tissue edema	SVM、LRA	Tumour volume >150 mL and maximum axial diameter >7.0 cm could be used as an independent predictor (multivariable analysis, P-value = 0.025, 0.045)
MRI-based radiomics	J. Zhong (10)	2022	N = 144	1	nnU-Net	Use FeAture Explorer 0.3.6 software (based on Pyradiomics 3.0)	Nomogram	radiomics models achieved AUC values of 0.699, 0.759 and 0.784
multimodal MRI	K. Y. Teo (28)	2022	N = 15	1	Manual segmentation	Extract statistical parameters and Haralick texture features	RF、LR、Multi-feature Fuzzy Clustering Technique、Weighted Majority Rule	The machine learning model combined with multimodal MRI showed good performance in predicting tumor necrosis, with AUC values of 0.999 in the training set and 0.915 in the test set
IVIM-MRI	Esha Baidya Kayal (29)	2022	N = 35	1	Manual segmentation	The BETV method was used for parameter estimation; Histogram analysis of the above parameters was performed	Independent sample t-test、Univariate and multivariate Cox regression analyses、Kaplan-Meier	The IVIM parameters showed AUC = 0.87, sensitivity = 86%, specificity = 77% at baseline (t0) and AUC = 0.96, sensitivity = 86%, specificity = 100% after the first chemotherapy cycle (t1)
MRI	J. Dufau (30)	2019	N = 69	1	Manual segmentation	Matlab	SVM、LDA、holdout	The analysis focused on the MRIs of 69 patients, 55.1% (38/69) of whom were good histological responders. The model obtained by support vector machines from initial MRI radiomic data had an AUROC of 0.98, a sensitivity of 100% (IC 95% [100%–100%]) and specificity of 86% (IC 95% [59.7%–111%]). DISCUSSION: Radiomic based on MRI data would predict the chemotherapy response before treatment initiation, in patients treated for osteosarcoma
MRI	G. J. Djuričić (31)	2017	N = 22	1	Manual segmentation (ImageJ marks ROI)	fractal analysis; Gray level co-occurrence matrix (GLCM) analysis	Bootstrap、X-tile 3.6.1	computational morphological analysis of primary osteosarcoma MR images using fractal and GLCM algorithms can predict chemotherapy response with high accuracy, as indicated by a ROC AUC of 0.82 and an accuracy of 82% in predicting actual chemotherapy outcomes
DCE-MRI	Zeng Yan-Ni (32)	2022	N = 25	1	Manual segmentation (Cover areas of the lesion that are most markedly enhanced and avoid areas of necrosis and blood vessels)	Semi-quantitative parameters of ROIs were automatically generated by software	Mann-Whitney U test	At the thresholds of 3.2%/s (Slope), 175 s (TTP) and 5.4% (ER), the sensitivity and specificity for predicting a good response to chemotherapy were 83.3% and 92.3%, 91.7% and 69.2%, 84.6% and 75.0%, respectively
DCE MRI and IVIM-DWI	Xibin Xia (33)	2022	N = 163	1*	Manual segmentation	Matlab	ANN、SVM、Combined feature selection methods (ReliefF and t-test)	After two treatment cycles, Ktrans, Kep and Ve values in CR/PR group were significantly lower than those in SD and PD groups, while D, ADC and f values were significantly higher in CR/PR group than in SD or PD group. ALP and LDH are positively correlated with Ktrans, Kep and Ve values, but negatively correlated with D, ADC and f values
T1CE-MRI	A. Bouhamama (19)	2022	N = 176	3	Manual segmentation	DCE-MRI parameters (KtransKtrans, KepKep, VeVe) and IVIM-DWI parameters (DD, D∗D∗, ADC, ff)	ReliefF and t-test、ANN、SVM、Logistic Regression	The model had an area under the ROC curve (AUC) of 0.95 and 0.97, respectively, with a sensitivity of 91% and a specificity of 92%
DCE-MRI	L. Zhang (34)	2021	N = 102	1	Manual segmentation (Exclude edema and vascular areas)	Radcloud; LASSO	KNN、SV、LR、Gridsearch algorithm、Nomogram	Models combining clinical risk factors (such as surgical stage) and radiomic features showed better predictive performance than radiomic models alone in both the training and test sets, with prediction accuracy (ACC) of 0.91 for the training set, ACC of 0.90 for the test set, and area under the ROC curve (AUC) of 0.94 and 0.95, respectively
Multiparametric MRI with DWI	M. M. Saleh (9)	2020	N = 53	1*	Manual segmentation	Apparent diffusion coefficient (ADC)、Minimum and average ADC values	0	The article highlights the importance of using multi-parameter MRI, especially DWI and ADC maps, in predicting the sensitivity of osteosarcoma to chemotherapy, and points out the limitations of traditional assessment methods based on tumor volume change
(18)F-FDF PET/CT								
(18)F-FDF PET/CT	B. C. Kim (35)	2021	N = 52	1	Manual segmentation (ROI)	LiFEx (version 4.0)	RF、GB、CNN	The chemotherapy response and metastasis test accuracy with image texture features was 0.83 and 0.76, respectively. The highest test accuracy and AUC of chemotherapy response with AUC_max, KI67, and EZRIN were estimated to be 0.85 and 0.89, respectively. The highest test accuracy and AUC of metastasis with AUC_max, KI67, and EZRIN were estimated to be 0.85 and 0.8, respectively. The metastasis prediction accuracy increased by 10% using radiogenomics data
(18)F-FDG PET/CT	Kim, J (36).	2021	N = 105	1	Manual segmentation (ROI)	LIFEx version 4.0	RF、SVM、2D CNN、dropout	The prediction model for NAC response with baseline PET0 ((18)F-FDG positron emission tomography/computed tomography (PET/CT) images were acquired before) texture features machine learning estimated a poor outcome, but the 2D CNN network using (18)F-FDG baseline PET0 images could predict the treatment response before prior chemotherapy in osteosarcoma. Additionally, using the 2D CNN prediction model using a tumor center slice of (18)F-FDG PET images before NAC can help decide whether to perform NAC to treat osteosarcoma patients
(18)F-FDG PET/CT	H. Song (37)	2019	N = 35	1	Manual segmentation (ROI)	PyRadiomics; calculate the traditional parameters such as SUVmax, SUVmean, MTV and TLG	Pearson correlation analysis、Student’s t-test、Kaplan-Meier	Metabolic tumor volume (MTV) is the best parameter for predicting chemotherapy response, with an area under ROC curve of 0.918 and a p value of less than 0.0001, indicating high predictive accuracy
(18)F-FDG PET/CT	S. Y. Jeong (38)	2019	N = 70	1	Manual segmentation (ROI)	Chang-Gung Image Texture Analysis tool box (open source software package based on MATLAB)	SVM、RF、GB、PCA	AUCs of the baseline (18)F-FDG features SUVmax, TLG, MTV, 1st entropy, and gray level co-occurrence matrix entropy were 0.553, 0538, 0.536, 0.538, and 0.543, respectively. However, AUCs of the machine learning features linear SVM, random forest, and gradient boost were 0.72, 0.78, and 0.82, respectively
99m Tc-MDP bone scintigraphy and 18F-FDG PET/CT	I. Lee, B (39)	2018	N = 62	1	Manual segmentation: 99m Tc-MDP bone scintigraphy (ROI)、 {18}F-FDG PET (VOI)	T/N (T/NT max)、SUVmax	Spearman rank correlation analysis	The Tc-MDP bone scan and F-FDG PET scan showed respective advantages with differing features
(18)F-FDG PET/CT	J. C. Davis (40)	2018	N = 34	1*	Manual segmentation	SUVmax、MRI:Tumor volume	logistic regression analysis	SUV (max) on routine images at 5 or 10 weeks and percentage change in SUV (max) from baseline to week 10 were metabolic predictors of a histologic response in OS
dual-phase (18)F-FDG PET/CT	B. H. Byun (41),	2015	N = 34	1*	Manual segmentation (ROI)	SUVmax、arly/delayed SUVmax change (RImax), early/delayed SUVmean change (RImean)、Percentage change before and after treatment (% SUV)	Mann-Whitney test、Wilcoxon signed rank test	By using combined criterion of %SUV and RImax2 or SUV2 and RImean1 or SUV2 and RImax2, accuracies were 81%, 77%, and 77%。The histological response after NAC could be predicted by using RImean1 before the initiation of NAC in osteosarcoma. The combined use of SUV and RI values may provide a better prediction
Combine multiple methods								
X-ray and MRI	Z. Luo (23)	2023	N = 102	1	Manual segmentation (ITK-SNAP marks ROI)	Pyradiomics package extract features from ROI Intra-group correlation coefficient (ICC) (ICCs >0.75):filter features mRMR and LASSO regression:feature selection	LR、DCA	Models using clinical, X-ray radiomics, MRI radiomics, X-ray plus MRI radiomics, and all data combined had AUC values of 0.760, 0.706, 0.751, 0.796, and 0.828, respectively
Multiparametric MRI with DWI	M. M. Saleh (9)	2020	N = 53	1*	Manual segmentation	Apparent diffusion coefficient (ADC)、Minimum and average ADC values	0	The article highlights the importance of using multi-parameter MRI, especially DWI and ADC maps, in predicting the sensitivity of osteosarcoma to chemotherapy, and points out the limitations of traditional assessment methods based on tumor volume change
99m Tc-MDP bone scintigraphy and 18F-FDG PET/CT	I. Lee, B (39)	2018	N = 62	1	Manual segmentation: 99m Tc-MDP bone scintigraphy (ROI)、 {18}F-FDG PET (VOI)	T/N (T/NT max)、SUVmax	Spearman rank correlation analysis	The Tc-MDP bone scan and F-FDG PET scan showed respective advantages with differing features
(18)F-FDG PET/CT	J. C. Davis (40)	2018	N = 34	1*	Manual segmentation	SUVmax、MRI:Tumor volume	logistic regression analysis	SUV (max) on routine images at 5 or 10 weeks and percentage change in SUV (max) from baseline to week 10 were metabolic predictors of a histologic response in OS

T2WI:axial T2-weighted imaging; T1CE:contrast-enhanced T1-weighted imaging; IVIM-MRI:Non-invasive intravoxel incoherent motion MRI; DCE-MRI:Dynamic Contrast Enhanced MRI; (18)F-FDG PET: (18)F-fluorodeoxyglucose positron emitted tomography; 1* = a single center prospective study; 1 = a singlecenter retrospective study; 2 = wo centers and different hospitals; 3 = in three different centers、Multicenter retrospective study; 0:No machine learning methods were used to predict chemotherapy effects, traditional statistical analysis was used; 0:No machine learning methods were used to predict chemotherapy effects, traditional statistical analysis was used; GB:Gradient Boosting algorithm; RF:Random Forest; LR:Logistic Regression; ROC:Receiver Operating Characteristic; DCA:Decision Curve Analysis; MLP:Multilayer Perceptron; LR:Logistic Regression; SVM:Support Vector Machine; DLR:Deep Learning Radiomics; BETV:Bi-exponential model with Total Variation Penalty function; GLCM:Gray Level Cooccurrence Matrix; SFR:Space-Filling Ratio; 2D CNN:2-dimensional convolutional neural network; LASSO:Least Absolute Shrinkage and Selection Operator; LOOCV:Leave-One-Out Cross-Validation; mRMR:Maximum Relevance Minimum Redundancy Feature Selection Method; ANN:Artificial Neural Network; KNN:K-nearest neighbor; CNN:Convolutional Neural Network.

### Quality evaluation

We employed the Radiomics Quality Score (RQS) tool to systematically evaluate study quality across critical research components. The assessment focused on: imaging acquisition protocols, including equipment parameters and scanning standardization to ensure data reliability; tumor segmentation methodology, evaluating accuracy and reproducibility as these directly impact feature extraction precision; and feature selection and computation processes, examining methodological rigor. This comprehensive scoring system enabled objective quality assessment across studies, helping identify methodological strengths and limitations while providing readers with transparent quality benchmarks (Tables 2, 3).

**TABLE 2:** RQS rating per study.

Study (Author, Year)		1	2	3	4	5	6	7	8	9	10	11	12	13	14	15	16	17	18	19	20	21	22	23	24	25	
		F. Yang2024	F. Zheng2024	Y. Zhang2024	T.Kanthawang2024	Z. Luo2023	D. Fu2023	J. Zhong2022	K. Y. Teo2022	Esha Baidya Kayal2022	Zeng Yan-Ni2022	Xibin Xia2022	A. Bouhamama2022	L. Xu2021	B. C. Kim2021	Kim, J.2021	L.Zhang2021	M. M. Saleh2020	C. Wu2019	H. Song2019	S. Y. Jeong2019	J. Dufau2019	I. Lee, B2018	J. C. Davis2018	G. J. Djuričić2017	B. H. Byun,2015	Median
Total 16 items (ideal score 36 = 100%)		16	15	16	15	10	6	10	6	8	8	6	7	15	9	9	9	9	9	10	7	9	10	7	−5	9	9
1	Image protocol quality (2 points)	2	2	2	2	1	1	1	1	2	0	1	1	1	2	1	1	1	1	1	1	1	1	2	1	2	1
2	Multiple segmentations (1 point)	1	1	1	1	0	0	0	0	0	0	0	0	1	0	1	1	1	0	0	1	0	0	0	0	1	0
3	Phantom study on all scanners (1 point)	0	0	0	0	0	0	0	0	0	0	0	0	0	0	0	0	0	0	0	0	0	0	0	0	0	0
4	Imaging at multiple time points (1 point)	1	0	1	0	0	0	0	0	1	0	0	0	0	0	1	1	1	0	0	1	0	1	1	0	1	0
5	Feature reduction or adjustment for multiple testing (−3 or 3 points)	3	3	3	3	−3	3	−3	3	−3	−3	3	3	3	3	−3	−3	−3	−3	−3	−3	−3	−3	−3	−3	−3	−3
6	Multivariable analysis with non-radiomics features (1 point)	1	1	1	1	1	1	1	1	1	0	1	1	1	1	1	1	1	0	1	1	0	0	0	0	0	1
7	Detect and discuss biological correlates (1 point)	0	0	0	0	0	0	0	0	0	0	0	1	0	1	0	0	0	0	0	0	0	0	0	0	0	0
8	Cut-off analyses (1point)	1	1	1	1	1	0	1	0	1	1	0	0	1	1	1	1	1	1	1	1	1	1	1	1	1	1
9	Discrimination statistics (2 points)	2	2	2	2	2	1	2	1	2	2	1	1	2	2	1	1	1	2	2	1	2	2	2	1	2	2
10	Calibration statistics (2 points)	1	1	1	1	2	1	2	1	0	2	1	1	0	0	0	0	0	2	2	0	2	2	0	0	0	1
11	Prospective study registered in a trial database (7 points)	0	0	0	0	0	0	0	0	7	0	0	0	0	0	0	0	0	0	0	0	0	0	7	0	0	0
12	Validation (−5, 2, 3, 4, or 5 points)	2	2	2	2	2	−5	2	−5	−5	2	−5	−5	2	−5	2	2	2	2	2	2	2	2	−5	−5	3	2
13	Comparison to gold standard (2 points)	0	0	0	0	2	2	2	2	0	2	2	2	2	2	2	2	2	2	2	0	2	2	0	0	0	2
14	Potential clinical utility (2 points)	2	2	2	2	2	1	2	1	2	2	1	1	2	2	2	2	2	2	2	2	2	2	2	0	2	2
15	Cost-effectiveness analysis (1 point)	0	0	0	0	0	1	0	1	0	0	1	1	0	0	0	0	0	0	0	0	0	0	0	0	0	0
16	Open science and data (0–4 points)	0	0	0	0	0	0	0	0	0	0	0	0	0	0	0	0	0	0	0	0	0	0	0	0	0	0

**TABLE 3:** Radiomics Quality Score (RQS) rating of included studies.

16 items		Range	Median	Percentage of ideal score, n (%)	Adherence rate, n (%)
Total 16 items (ideal score 36 = 100%)		—8–36	9	244/900 (27.1)	220/400 (55)
1	Image protocol quality (2 points)	0–2	1	16/50 (32)	24/25 (96)
2	Multiple segmentations (1 point)	0–1	0	10/25 (40)	10/25 (40)
3	Phantom study on all scanners (1 point)	0–1	0	0/25 (0)	0/25 (0)
4	Imaging at multiple time points (1 point)	0–1	0	10/25 (40)	10/25 (40)
5	Feature reduction or adjustment for multiple testing (−3 or 3 points)	−3 to 3	−3	30/75 (40)	10/25 (40)
6	Multivariable analysis with non-radiomics features (1 point)	0–1	1	18/25 (72)	18/25 (72)
7	Detect and discuss biological correlates (1 point)	0–1	0	2/25 (8)	23/25 (92)
8	Cut-off analyses (1point)	0–1	1	22/25 (88)	22/25 (88)
9	Discrimination statistics (2 points)	0–2	2	32/50 (64)	25/25 (100)
10	Calibration statistics (2 points)	0–2	1	14/50 (28)	15/25 (60)
11	Prospective study registered in a trial database (7 points)	0–7	0	14/175 (8)	2/25 (8)
12	Validation (−5, 2, 3, 4, or 5 points)	−5 to 5	2	0/125 (0)	17/25 (68)
13	Comparison to gold standard (2 points)	0–2	2	32/50 (64)	16/25 (64)
14	Potential clinical utility (2 points)	0–2	2	40/50 (80)	24/25 (96)
15	Cost-effectiveness analysis (1 point)	0–1	0	4/25 (16)	4/25 (16)
16	Open science and data (0–4 points)	0–4	0	0/100 (0)	0/25 (0)

The ideal score was described as score and percentage of score to ideal score for each item. In the cases where a score of one point per item was obtained, the study was considered to have basic adherence to each item. The adherence rate was calculated as proportion of the number of articles with basic adherence to number of total articles.

The RQS evaluation served dual purposes: guiding researchers in improving study design and enabling readers to critically appraise result reliability. All quality assessments were conducted independently by two reviewers, with discrepancies resolved through consensus discussion involving a third investigator when necessary. This rigorous approach ensured unbiased quality appraisal while maintaining consistency with current best practices in radiomics research.

### Statistical analysis

**FIGURE 2:**
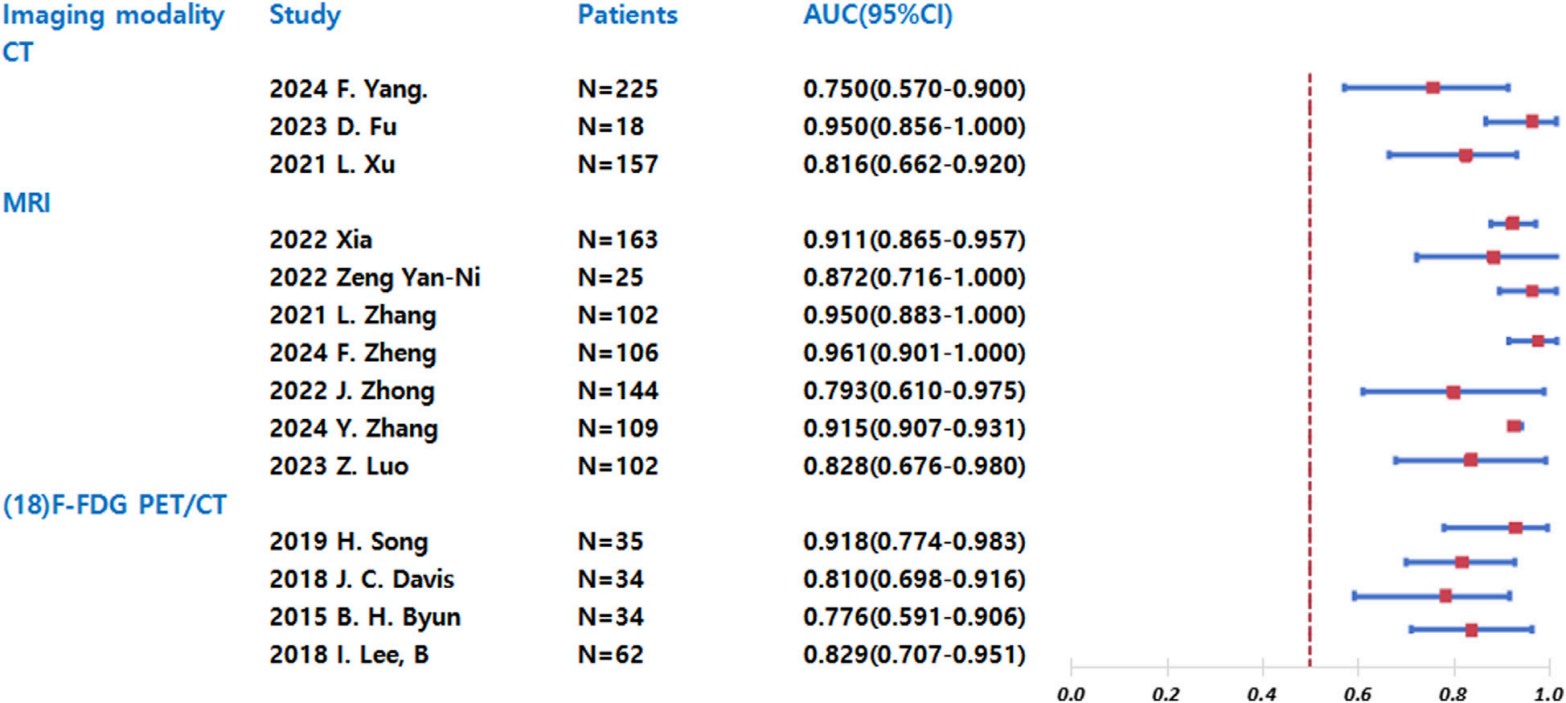
Forest plot of AUC values for chemotherapy response prediction across imaging modalities.

**FIGURE 3:**
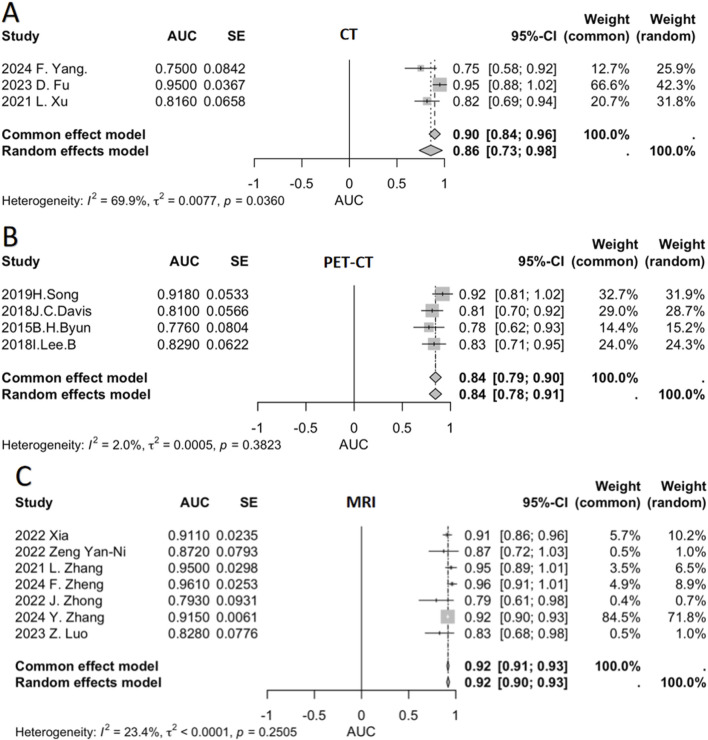
Pooled AUC values across imaging modalities.

To quantitatively evaluate the predictive performance of different imaging modalities (Xray, CT, MRI, PET-CT) in assessing osteosarcoma response to neoadjuvant chemotherapy, we conducted a meta-analysis using a random-effects model. Pooled sensitivity, specificity, and area under the receiver operating characteristic curve (AUC) were calculated to determine the diagnostic accuracy of each imaging approach. The I^2^statistic was used to assess study heterogeneity, with significant heterogeneity defined as I^2^ > 50% and p < 0.05. All statistical analyses were performed using specialized software (Rstudio, IBM SPSS Statistics, Review Manager) to ensure accuracy and reliability (Figures 2, 3).

This analytical approach allowed for robust comparison of imaging modalities while accounting for potential variations across studies, thereby strengthening the validity of our findings.

### Radiomics and deep learning methodology

The radiomics pipeline for osteosarcoma NAC prediction relies heavily on deep learning (summarized in Table 1) (10, 17, 19).

1. Image segmentation: Frameworks like nnU-Net and Auto3DSeg enable precise tumor delineation;
2. Feature extraction: Software tools extract thousands of quantitative features (morphological, intensity-based, textural) from MRI/CT to characterize tumor heterogeneity;
3. Model building: CNNs automate hierarchical feature learning, bypassing manual selection bias. Their end-to-end training—via localized convolution kernels and backpropagation-optimizes predictive performance for high-dimensional imaging data. These models now extend beyond chemosensitivity prediction to tumor grading, survival analysis, and metastatic risk stratification.

## Results

### Literature retrieval and study selection

A total of 1015 potentially relevant records were initially identified through searches in three databases: PubMed (n = 257), Embase (n = 632), and Web of Science (n = 126). After removing duplicate records, 423 studies remained for further evaluation.

Subsequently, two independent investigators screened the titles and abstracts of these 423 studies, excluding 321 records that clearly did not meet the eligibility criteria. Additionally, 6 reports could not be retrieved despite efforts to access full texts, leaving 96 studies for full - text review.

During the full - text assessment, studies were excluded for the following reasons: no outcome of interest (n = 3), lack of osteosarcoma - specific results (n = 3), focus only on post - neoadjuvant chemotherapy (NAC) assessment (n = 4), focus only on prognosis rather than prediction (n = 11), evaluation of chemotherapy response without predictive analysis (n = 13), duplicate articles (n = 4), and no association with radiomics (n = 33). Finally, 25 studies were included in the systematic review and meta - analysis. The detailed flow of study selection is presented in Figure 1.

### Statistical analysis

The forest plot (Figure 2) presents a comparative analysis of diagnostic performance among different imaging modalities (CT, MRI, and ^18^F-FDG PET/CT) in predicting chemotherapy response sensitivity. Chemotherapy response sensitivity was defined as the histological response of the tumor to NAC, with a good response defined as ≥90% tumor necrosis. All included studies used this consistent cutoff. For each modality, we have included studies from various years and authors, along with their respective sample sizes. The primary outcome measure-the area under the curve (AUC) with 95% confidence intervals-serves as an indicator of diagnostic accuracy, where values closer to 1.0 represent superior performance. Key findings from the analysis reveal distinct patterns across modalities: CT demonstrated moderate diagnostic utility with AUC values ranging from 0.750 to 0.950 across studies, though with notable variability between reports. MRI consistently showed high and stable predictive accuracy, with AUC values between 0.873 and 0.961 in all included studies. ^18^F-FDG PET/CT exhibited the widest performance variation, with AUC values spanning from 0.726 to 0.948, indicating inconsistent predictive capability across different study populations.

The comparative analysis suggests that MRI currently offers the most reliable and consistent performance for predicting chemotherapy response sensitivity in osteosarcoma, while both CT and ^18^F-FDG PET/CT show greater variability in diagnostic accuracy. These findings may inform modality selection for clinical decision-making regarding neoadjuvant chemotherapy response assessment.

We conducted a meta-analysis to pool AUC data from multiple relevant studies (Figure 3). This figure presents the AUC values of individual studies for different imaging modalities (CT, PET-CT, MRI), along with their respective weights and the pooled AUC values calculated using both fixed-effect and random-effects models. This comprehensive approach evaluates the diagnostic performance of each modality in predicting chemotherapy response sensitivity.

For CT (Figure 3A), three studies were included, showing variability in AUC values—ranging from 0.750 (F.Yang, 2024) to 0.950 (D.Fu, 2023). Significant heterogeneity was observed (I^2^ = 69.9%), indicating limited consistency across studies. The pooled AUC was 0.90 (fixed-effect) and 0.86 (random-effects).

PET-CT (Figure 3B) incorporated four studies, with AUC values fluctuating between 0.7760 [Byun, (41)] and 0.9180 [Song, (37)]. Low heterogeneity (I^2^ = 2.0%) suggested high inter-study consistency. Both models yielded a pooled AUC of 0.84.

MRI (Figure 3C) demonstrated the strongest performance among the three modalities. Seven studies were analyzed, with most reporting high and clustered AUC values {e.g., 0.9410 [Xia, (33)] and 0.8280 [Luo, (23)]}. Heterogeneity was low (I^2^ = 23.4%), and the pooled AUC was 0.92 under both models.

MRI’s superiority stems from several factors. First, its exceptional soft-tissue contrast and spatial resolution enable precise visualization of tumor involvement in bone marrow, soft tissues, neurovascular bundles, and joints—critical for chemotherapy response assessment (42). Second, radiomics allows high-throughput feature extraction from MRI data, while multimodal sequences integrate complementary biological information to enhance predictive accuracy (43, 44). Advanced techniques like IVIM-MRI further contribute to this capability (29).

Additionally, functional MRI methods such as DCE-MRI and DWI quantify tumor vascularity and cellularity, providing robust biomarkers for treatment response (9). Baseline MRI features also show significant value in predicting chemoresistance, particularly when combined with machine learning (45). Unlike CT, MRI avoids ionizing radiation and offers multiplanar imaging (axial, coronal, sagittal), facilitating comprehensive anatomic evaluation. These advantages make MRI indispensable for pretreatment tumor staging and chemotherapy response prediction, ultimately supporting clinical decision-making (46).

## Discussion

The present review confirms that radiomics, particularly when integrated with deep learning, holds significant promise for predicting NAC response in osteosarcoma. Among imaging modalities, MRI demonstrates superior performance, with pooled AUC of 0.92 across studies, making it the most reliable tool for clinical decision-making. This is attributed to its ability to capture both anatomical and functional tumor characteristics (e.g., DWI for cellularity, DCE-MRI for vascularity), which directly reflect chemotherapy-induced biological changes.

We categorized studies by imaging type and generated a pooled AUC forest plot (Figure 3), highlighting sensitivity differences.

### X-ray films

Plain radiography serves as a fundamental imaging modality for the initial evaluation of suspected bone tumors. By assessing lesion location, mineralization patterns, and margin characteristics, it provides essential guidance for differential diagnosis and remains the optimal macroscopic imaging method for demonstrating primary bone tumor features. Prior to advanced imaging (CT or MRI), radiologists should review radiographs to optimize scan protocols based on tumor size and location, ensuring maximal diagnostic yield from subsequent examinations (42).

In the context of osteosarcoma NAC response prediction, radiography maintains critical clinical relevance. Emerging evidence demonstrates that integrating radiographic features with clinical and advanced imaging data significantly enhances predictive accuracy. A 2023 study by Luo et al (23) analyzed combined X-ray and MRI features in 102 osteosarcoma patients. Their results revealed that a multimodal model incorporating radiomics and clinical parameters achieved superior performance (AUC = 0.828) compared to: Clinical-only models (AUC = 0.760); X-ray-only models (AUC = 0.706); MRI-only models (AUC = 0.751); Simple X-ray + MRI combined models (AUC = 0.796).

These findings underscore that while radiography has inherent limitations as a standalone predictor of NAC response, its foundational imaging data—when systematically integrated into comprehensive assessments—provides indispensable value for refining predictive accuracy and supporting personalized treatment strategies.

### Computed tomography

CT imaging serves as a cornerstone in osteosarcoma diagnosis and treatment evaluation, providing precise assessment of tumor volume dynamics, local extension, and critical morphological features including margin characteristics, internal architecture, calcification patterns, bone destruction extent, and periosteal reaction (43). The advent of radiomics has further enhanced CT’s utility by enabling quantitative extraction of subtle imaging biomarkers to improve chemotherapy response prediction.

Extensive research has demonstrated the significant efficacy of CT-derived analyses in predicting osteosarcoma chemotherapy response through multiple sophisticated approaches. These methodologies range from radiomics model construction to the integration of clinical parameters and peri-tumoral bone characterization.

The 2021 study by Xu (15) revealed critical insights into feature selection strategies. Their analysis demonstrated that incorporating both tumor and peri-tumoral bone features significantly improved pathological response prediction accuracy compared to tumor-only analysis. Using leave-one-out cross-validation (LOOCV), the combined-feature classifier achieved AUC values of 0.791 (95% CI: 0.706–0.860) in the training set and 0.816 (95% CI: 0.662–0.920) in the validation set. Further enhancement was observed when clinical parameters including age, sex, and tumor location were integrated, boosting the validation AUC to 0.811.

Subsequent investigations have identified additional potent CT biomarkers. Fu et al. (25) study of pediatric osteosarcoma patients identified two exceptional predictors: the peri-tumoral fat attenuation index (FAI) demonstrated perfect sensitivity (100%) with an AUC of 0.950, while 6-h methotrexate serum concentration showed perfect specificity (100%) with an AUC of 0.963. These findings suggest complementary roles for imaging and pharmacological metrics in response prediction.

The evolving field of radiomics received further validation through F. Yang’s 2024 work (24), which distilled 1,233 high-consistency CT features to three clinically relevant biomarkers via LASSO logistic regression. While the standalone radiomics model showed moderate performance (validation AUC = 0.68), its integration with clinical factors improved predictive accuracy (validation AUC = 0.75). The resultant nomogram exhibited excellent calibration between predicted and observed Huvos grades, confirming the clinical utility of combined models.

These cumulative findings underscore CT imaging’s dual role in osteosarcoma management providing both detailed anatomical evaluation and quantitative biomarkers for treatment response prediction. The consistent demonstration of AUC values exceeding 0.75 across multiple study designs confirms CT’s robust predictive value when combined with advanced analytical approaches, offering clinicians valuable tools for personalized therapeutic decision-making.

The progressive improvement in predictive accuracy from isolated radiomics (AUC ≈ 0.70) to integrated models (AUC ≈ 0.80) highlights the importance of multimodal data synthesis, with CT serving as a fundamental component in comprehensive assessment protocols.

### Magnetic resonance imaging

Magnetic resonance imaging (MRI) has emerged as a cornerstone in osteosarcoma management, offering unparalleled capabilities in predicting neoadjuvant chemotherapy (NAC) response through its multimodal imaging approach. Conventional T1-and T2-weighted sequences provide exceptional soft tissue contrast, enabling precise delineation of tumor boundaries, intramedullary extension,and soft tissue component size (42). These anatomical assessments are significantly enhanced by functional MRI techniques: DCE-MRI (47) quantifies tumor vascular permeability and perfusion characteristics, while DWI evaluates cellular density-both serving as critical biomarkers for chemotherapy response monitoring (44, 46).

The predictive superiority of MRI manifests in several key aspects:

#### Superior anatomical and functional assessment

MRI’s high spatial resolution and multiplanar capabilities allow comprehensive evaluation of neurovascular bundle involvement and joint infiltration, establishing crucial baseline data for treatment planning. Numerous studies validate MRI’s effectiveness in tracking tumor aggressiveness, volumetric changes, and structural alterations during therapy (43). The inherent contrast between tumor and normal tissue on T1/T2-weighted imaging provides reliable morphological assessment (42), while functional sequences offer dynamic monitoring: DCE-MRI detects chemotherapy-induced vascular changes, and DWI demonstrates particular utility in differentiating responders from non-responders at mid-treatment (9). Saleh et al. (9) emphasized the prognostic value of DWI-derived apparent diffusion coefficient (ADC) measurements, noting significant ADC increases in responders and establishing ADC percentage change as a robust response indicator.

#### Advanced radiomics and predictive modeling

MRI’s multiparametric nature has propelled its dominance in radiomics research (44). The 2022 study by Zhong et al. (10) developed an automated pipeline using nnU-Net segmentation (Dice coefficient = 0.869) to extract radiomic features from preoperative T2-weighted images. Their clinical-radiomic nomogram achieved superior performance (AUC = 0.793, accuracy = 79.1%) compared to clinical-only (AUC = 0.699) or radiomics-only (AUC = 0.759) models. Subsequent research by Zhang et al. (2024) (20) demonstrated even more impressive results, with a combined pre- and post-NAC model reaching AUC values of 0.999 (training) and 0.915 (testing). Zheng et al. (27) deep learning radiomics (DLR) approach further advanced the field, achieving 93.8% accuracy and 0.961 AUC in NAC response prediction, outperforming conventional models.

#### Multimodal integration and specialized techniques

The synergistic combination of MRI sequences provides multidimensional tumor characterization. Some studies used multimodal MRI before neoadjuvant chemotherapy to build machine learning models, and achieved high AUC values in the training set and test set (0.999 and 0.915, respectively) (28). Multiparametric models incorporating DCE-MRI and intravoxel incoherent motion (IVIM) DWI have shown particular promise, with parameters like D-value and Kep demonstrating strong predictive value for disease progression (33). Specialized analysis methods, including fractal analysis and gray-level co-occurrence matrix (GLCM) calculations, have identified novel biomarkers like the shape factor ratio (SFR) with 82% prediction accuracy (31). Analysis of pretreatment MRI parameters has identified several robust imaging biomarkers for predicting chemotherapy resistance in osteosarcoma patients. Among these, tumor volume and maximum axial diameter demonstrate statistically significant predictive value, with tumor size emerging as the most potent independent predictor (p = 0.025 for volume, p = 0.045 for maximum diameter) (11).

#### DCE-MRI’s unique contributions

As a functional MRI technique, DCE-MRI provides distinct advantages in chemotherapy response assessment. Its semi-quantitative parameters (slope, time-to-peak, enhancement ratio) have demonstrated remarkable predictive value, with Zeng et al. (32) reporting 83.3%–91.7% sensitivity and 69.2%–92.3% specificity at optimal thresholds. The radiomics model based on DCE-MRI data established by machine learning has achieved excellent performance. One study reported that the auc of training/testing was 0.94/0.95 and the accuracy was more than 90% (34). Another pre-treatment model incorporating DCE-MRI features reached AUCs of 0.95 (training) and 0.97 (validation) with 91% sensitivity and 92% specificity (19). These results surpass conventional imaging modalities in functional assessment capability.

The accumulated evidence positions MRI as an indispensable tool for personalized osteosarcoma management. Its ability to integrate high-resolution anatomical imaging with functional and radiomic biomarkers creates a comprehensive assessment platform for chemotherapy response prediction. As deep learning technology continues to improve acquisition protocols and analysis methods, MRI’s role in optimizing treatment strategies and improving patient outcomes will undoubtedly expand (9). The modality’s non-invasive nature, absence of ionizing radiation, and capacity for longitudinal monitoring further solidify its position as the imaging technique of choice for osteosarcoma NAC response evaluation.

### Radionuclide bone imaging

Radionuclide bone scintigraphy remains a valuable imaging modality for osteosarcoma evaluation, particularly in detecting skeletal lesions that may be occult on conventional radiographs, though its specificity is limited by similar uptake patterns in both benign and malignant processes. This technique provides essential whole-body screening capability for multifocal disease detection and enables longitudinal monitoring of therapeutic response across multiple lesions (48). In parallel, advanced MRI techniques have revolutionized response assessment in osteosarcoma patients undergoing neoadjuvant chemotherapy, with the interval change in apparent diffusion coefficient (ADC) between baseline and mid-treatment MRI (ΔADC2) emerging as a particularly promising early response biomarker. ΔADC2 demonstrates superior accuracy to later ADC measurements in forecasting treatment outcomes, often revealing therapeutic effects weeks before morphological changes become apparent. The absence of favorable ΔADC2 changes at mid-treatment evaluation may help identify non-responders, potentially enabling earlier transition to alternative chemotherapy regimens or surgical planning adjustments while avoiding unnecessary treatment toxicity. These quantitative ADC changes reflect meaningful chemotherapy-induced alterations in tumor cellular density and tissue microstructure. The integration of radionuclide imaging’s whole-body disease burden evaluation with functional MRI’s precise quantitative monitoring creates a comprehensive assessment framework that supports more informed, personalized treatment decisions in osteosarcoma management (49).

### ^18^F-Fluorodeoxyglucose Positron Emission Tomography (^18^F-FDG-PET)

Research on FDG-PET for predicting neoadjuvant chemotherapy response in osteosarcoma has made significant progress. As early as the 1990s, initial studies explored the relationship between FDG-PET parameters and chemotherapy efficacy. Between 2010 and 2015, researchers found that a significant decrease in standardized uptake value (SUV) of osteosarcoma lesions on FDG-PET after chemotherapy correlated with better treatment response. In 2015, Byung Hyun Byun et al. (41) demonstrated that the mean SUV change (RImean1) in dual-phase 18F-FDG PET/CT (early and delayed imaging) before chemotherapy could predict favorable histological response, with an optimal threshold of <10%, sensitivity of 92%, specificity of 57%, and diagnostic accuracy of 71%.

Multiparametric FDG-PET analysis, including SUVmax, total lesion glycolysis (TLG), metabolic tumor volume (MTV), and total tumor glycolysis, has further improved the accuracy of predicting neoadjuvant chemotherapy response, providing stronger support for precision medicine. Predictive models based on 18F-FDG uptake heterogeneity features (e.g., gray-level size zone matrix, GLSZM) achieved an AUC of 0.626 at baseline (PET0) (36). Additionally, texture features such as coarse-grained neighborhood gray-tone difference matrix (NGTDM) were independent prognostic factors, significantly associated with event-free survival (p = 0.005), confirming the potential of baseline 18F-FDG PET texture analysis in predicting chemotherapy response (37).

For example, in 2019, Song et al. (37) identified MTV as the best predictor of chemotherapy response, with an AUC of 0.918 (p < 0.0001), indicating high predictive accuracy. In 2018, Lee et al. (39) found that both 99mTc-MDP bone scintigraphy and 18F-FDG PET/CT had high accuracy in predicting osteosarcoma treatment response, with no significant difference between the two (P = 0.44). For predicting good pathological response (≥90% tumor necrosis), 99mTc-MDP bone scintigraphy (D%T/NTmax) showed 83.3% sensitivity and 75.0% specificity, while 18F-FDG PET/CT (D%SUVmax) demonstrated 80.0% sensitivity and 81.3% specificity, suggesting their complementary role in clinical decision-making.

FDG-PET parameter changes at different treatment time points also have predictive value. Davis et al. (40) found that SUVmax at 5 weeks (P = 0.034) and 10 weeks (P = 0.022), as well as the percentage change in SUVmax from baseline to week 10 (P = 0.021), were strong predictors of pathological response, with sensitivities of 0.93, 0.93, and 0.79 and specificities of 0.53, 0.71, and 0.76, respectively. Early PET/CT assessment at 5 weeks could help adjust subsequent treatment strategies.

Recent breakthroughs involve integrating advanced technologies such as machine learning (e.g., linear SVM, random forest, gradient boosting) and deep learning (e.g., 2D CNN) with FDG-PET texture features to enhance predictive performance. In 2019, Jeong et al. (38) analyzed baseline 18F-FDG PET texture features in 70 high-grade osteosarcoma patients and found that machine learning models achieved AUCs of 0.72 (linear SVM), 0.78 (random forest), and 0.82 (gradient boosting). Changes in texture features post-chemotherapy, such as SUVmax percentage change (AUC = 0.863), first entropy (AUC = 0.767), and GLCM entropy (AUC = 0.775), also predicted treatment response. In 2021, Kim et al. (36) reported that a 2D CNN deep learning model outperformed traditional machine learning in pre-chemotherapy prediction, with test accuracy ranging from 0.625 to 0.760.

Radiogenomics, combining imaging features with gene expression (e.g., KI67 and EZRIN), further improved predictive accuracy. A random forest model achieved a test accuracy of 0.83, which increased to 0.85 (AUC = 0.89) when integrated with AUC_max, KI67, and EZRIN (35).

Additionally, 99mTc-MIBI scintigraphy is a useful tool for assessing neoadjuvant chemotherapy response in osteosarcoma. A 2019 study by Wu et al. (26) on 30 osteosarcoma patients showed that pre-chemotherapy tumor washout rate negatively correlated with necrosis rate, while post-chemotherapy uptake change rate positively correlated with necrosis rate, confirming its predictive value.

Although FDG-PET has limitations in primary bone tumor diagnosis, its role in detecting non-osseous metastatic lesions provides critical information for tumor staging and treatment planning. With further multidisciplinary research, FDG-PET is expected to play an even greater role in optimizing osteosarcoma management.

From a clinical perspective, these findings can transform patient management in three key ways:

Treatment personalization: Preoperative radiomics models can identify patients unlikely to respond to standard NAC, enabling early switch to alternative therapies (e.g., targeted agents in clinical trials) and avoiding unnecessary toxicity (2, 19); Trial design optimization: By stratifying patients based on predicted response, radiomics can reduce sample sizes in clinical trials and improve the efficiency of novel therapy evaluation (10); Workflow integration: MRI radiomics can be incorporated into routine preoperative assessments, with automated segmentation and model prediction taking <30 minutes—compatible with standard clinical timelines (2, 19).

Despite these strengths, limitations exist. Most studies are single-center with small sample sizes, limiting generalizability (6, 50). Additionally, variability in imaging protocols (e.g., MRI sequence parameters) across institutions hinders model reproducibility (46). Future research should prioritize: Standardizing imaging acquisition and radiomics pipelines across centers to ensure model robustness; Validating models in prospective cohorts, with focus on real-world clinical utility (e.g., reducing unplanned surgeries due to poor response); Integrating radiomics with genomic and clinical data to develop “multi-omics” prediction systems, further refining patient stratification (5, 35).

In summary, radiomics-driven prediction of NAC response in osteosarcoma is transitioning from a research tool to a clinically actionable technology. With rigorous validation and standardization, it will play a central role in personalized oncology, improving patient outcomes and optimizing healthcare resource allocation (6, 50).

## Conclusion

Predicting chemotherapy response in osteosarcoma remains a critical challenge for optimizing treatment and improving outcomes. While conventional imaging techniques—X-ray, CT, MRI, bone scintigraphy, and FDG-PET—each provide valuable insights, limitations persist. MRI shows particular promise in assessing neoadjuvant chemotherapy response, whereas FDG-PET offers metabolic profiling advantages.

Current limitations include variability in imaging protocols, analytical methods, and the predominance of single-center studies with small sample sizes, which may affect generalizability. Moving forward, standardizing imaging protocols, expanding multicenter collaborations, and refining deep learning models will be essential. Incorporating clinicopathological and molecular data may further improve predictive performance. Future research should focus on integrating novel biomarkers, artificial intelligence, and multimodal imaging to enhance predictive accuracy and enable personalized treatment strategies. With these advancements, more precise and clinically actionable predictive systems can be developed, ultimately improving osteosarcoma management and patient outcomes.

## Data Availability

The original contributions presented in the study are included in the article/supplementary material, further inquiries can be directed to the corresponding authors.

## Abbreviations

AI, Artificial intelligence; CI, Confidence interval; CLAIM, Checklist for Artificial Intelligence in Medical Imaging; DOR, Diagnostic odds ratio; NAC, Neoadjuvant chemotherapy; QUADAS-2, Modified Quality Assessment of Diagnostic Accuracy Studies; RQS, Radiomics Quality Score; SROC, Summary receiver operating characteristic; TRIPOD, Transparent Reporting of a multivariable prediction model for Individual Prognosis or Diagnosis.
